# Thyrotoxic periodic paralysis as the first presentation of Graves' disease: A case report

**DOI:** 10.1002/ccr3.7188

**Published:** 2023-05-14

**Authors:** Abdulrahman F. Al‐Mashdali, Mohammed Alfatih, Waseem Umer, Yasser Eldeeb

**Affiliations:** ^1^ Department of Internal Medicine Hamad Medical Corporation Doha Qatar; ^2^ Faculty of Medicine Alzaiem Alazhari University Khartoum Sudan; ^3^ Department of Infectious Diseases Hamad Medical Corporation Doha Qatar

**Keywords:** case report, Graves' disease, hyperthyroidism, thyrotoxic periodic paralysis

## Abstract

Thyrotoxic periodic paralysis (TPP) is a rare disease seen predominantly in men of Asian origin. It should be considered in the differential diagnosis of patients with acute onset of weakness, and it is reversible after the correction of serum potassium. TPP can rarely be the initial presentation of Graves' disease.

## Abstract 2

The clinician should investigate for hyperthyroidism in a patient presenting with thyrotoxic periodic paralysis, even in the absence of other features of thyrotoxicosis.

## 1 INTRODUCTION

Graves' disease is an autoimmune condition in which thyroid‐stimulating hormone receptor antibodies result in the overproduction of thyroid hormones, leading to thyrotoxicosis manifestations. It is the most common cause of hyperthyroidism worldwide.^1^ Patients with Graves' disease usually present with classic clinical manifestations, including but not limited to heat intolerance, palpitations, tremor, anxiety, and weight loss. However, rarely, some patients might initially present with unusual clinical manifestations.^2^, ^3^ Thyrotoxic periodic paralysis (TPP) is a potentially life‐threatening complication of thyrotoxicosis, and the attack is characterized by acute and reversible severe painless muscle weakness. Most patients presenting with TPP have been previously diagnosed with Graves' disease.^4^ Herein, we present an extremely rare case of Graves' disease initially presenting with TPP without other clinical features of hyperthyroidism.

## 2 CASE PRESENTATION

Twenty‐eight‐year‐old Nepalese male, previously healthy, presented to the emergency department with a 1‐day history of persistent bilateral lower limb weakness that started suddenly while at home and was associated with generalized muscle aches. There was no associated weakness in the upper limbs, numbness or paresthesia, dizziness, bowel or bladder incontinence, diplopia, or bulbar symptoms. He had no symptoms of thyrotoxicosis. He denied a history of heavy physical exertion, recent infection, or carbohydrate‐rich food consumption. He is a non‐smoker and non‐alcohol drinker.

On examination, he was afebrile, had a blood pressure of 135/80 mmHg, a heart rate of 76 beats/minute, a respiratory rate of 14 breaths/min, and maintained 99% oxygen saturation on the room air. A neurological examination revealed a Glasgow Coma Scale (GCS) of 15/15, intact cranial nerves, and normal motor and sensory examination of upper limbs. The lower limb motor examination revealed that the medical research council scale for muscle power was 1/5 bilaterally. Ankles and knees deep tendon reflexes were diminished bilaterally. Plantar reflexes were equivocal. Sensory examination and coordination were unremarkable. He had no exophthalmos, lid retraction, or lid lag. Neck examination revealed no goiter or thyroid bruit. Also, he has no hand tremors or leg edema. Cardiac, respiratory, and abdominal examinations were all unremarkable. Investigations (Table 1) revealed severe hypokalemia (potassium level of 2.2 mmol/L). The electrocardiogram (ECG) showed normal sinus rhythm without other remarkable findings.

**TABLE 1:** Relevant laboratory results in the hospital.

Variables	Results	Reference range
Wbc (10^3^/μL)	8500	4000–11,000
Hgb (g/dL)	14.8	13–17
Platelets count (10^3^/μL)	186	150–400
Urea (mmol/L)	5.2	2.5–7.9
Creatinine (μmol/L)	61	62–106
Na (mmol/L)	142	135–145
K (mmol/L)	2.2	3.5–5.5
Cl (mmol/L)	105	95–105
Bicarbonate (mmol/L)	24	22–28
Adjusted calcium (mmol/L)	2.47	2.20–2.60
Magnesium (mmol/L)	0.76	0.70–1.0
Albumin (g/L)	41	35–55
Total protien (g/L)	77	60–78
ALT (U/L)	16	8–20
AST (U/L)	17	8–20
Alkaline phosphatase (U/L)	127	40–129
Billirubin (total) (μmol/L)	7	2–17
CRP (mg/L)	3	0–5

Abbreviations: ALT, alanine aminotransferase; APTT, activated partial thromboplastin time; AST, aspartate aminotransferase; CRP, C‐reactive protein; Hgb, hemoglobin; INR, international normalization ratio; PT, prothrombin time; WBC, white blood cell.

Urgent potassium correction (potassium chloride infusion 40 mmol in 500 mL 0.9% normal saline) through the central line was initiated. Twenty‐four hours from admission, the serum potassium level reached 4.5 mmol/L (3.3–5.5 mmol/L). The patient's lower limb weakness dramatically improved after potassium correction. The thyroid function test was consistent with primary hyperthyroidism and positive TSH‐receptor (TSH‐R) autoantibodies, indicating Graves' disease (Table 2). The patient was started on carbimazole 10 mg oral three times daily and propranolol 10 mg once daily. In the 2‐month follow‐up, the patient was asymptomatic and did not report any further episodes of weakness or features of hyperthyroidism.

**TABLE 2:** Thyroid function test.

Variables	Results	Reference range
TSH (mIU/L)	<0.01	0.3–4.2
FT3 (pmol/L)	13.3	3.7–6.4
FT4 (pmol/L)	26.9	11–23.3
TSH R antibody (IU/L)	Positive	–
Anti TPO	Negative	–

Abbreviations: Anti TPO, anti thyroperoxidase antibody; TSH R antibody, thyroid‐stimulating hormone receptor antibody.

## 3 DISCUSSION

The present case describes a young Nepalese male suffering from thyrotoxic periodic paralysis as the first presenting manifestation of Graves' disease. TPP can appear with thyrotoxicosis of any origin, such as thyroiditis, toxic adenoma, or toxic nodular goiter. However, the majority of cases are due to Graves' disease.^4^ TPP is an uncommon condition and predominantly affects Asian males, including Chinese, Japanese, Vietnamese, Filipino, and Koreans, with an incidence of 2% in Asian populations with hyperthyroidism compared with 0.1%–0.2% in non‐Asian populations.^4^, ^5^, ^6^ Cases of TPP in African American males and Arabic ethnicity are rare.^6^, ^7^, ^8^ Even though hyperthyroidism is more common in women than men, with female to male ratio of 9:1. TPP is a condition that has been observed more frequently in males than in females (male‐to‐female ratio ranging from 17:1 to 70:1.^5^). The age of onset is usually between 20 and 40 years of age coinciding with the peak age for hyperthyroidism.^9^

The pathogenesis of TPP is not fully understood; however, it has been hypothesized that it may be caused by more androgen‐ which increases the activity of the Na^+^/K^+^‐ATPase pump or more pronounced hyper‐insulinemia in males. Moreover, higher sympathetic and catecholamine‐induced Na^+^–K^+^ ATPase activity in males and increased muscle mass in males have also been described as possible mechanisms. Na/K ATPase activity is also inhibited by estrogens and progesterone, which may explain the predominance of the disorder among men. Recent data indicate that TPP can be due to a combination of genetics, thyrotoxicosis, and environmental factors.^10^

There are several triggers for TPP, such as high carbohydrate ingestion, strenuous exercise, trauma, acute upper respiratory tract infection, diarrhea, high‐salt diet, emotional stress, exposure to cold, alcohol ingestion, menstruation, and use of drugs such as corticosteroids at high dose, antiretroviral for AIDS or interferon‐alpha, epinephrine, acetazolamide, and non‐steroidal anti‐inflammatory drugs have been reported.^10^, ^11^ Although the exact pathophysiology of TPP is still unclear, our patient was an Asian male, and there was no apparent precipitating factor for the episode.

Muscle weakness in TPP ranges from mild weakness to complete flaccid paralysis. Although a thyrotoxic state is associated with hyperreflexia, diminished or absent deep tendon reflexes are typically seen in TPP, and respiratory muscles, bulbar and ocular are rarely involved. Involvement of the sensory system, cognitive functions, and bladder has never been seen.^4^, ^5^ Recent data indicate that muscle weakness in TPP can be due to hypokalemia, hypophosphatemia, and hypomagnesemia.^9^

Hypokalemia in TPP occurs due to a massive shift of potassium into the cells, which has been linage to excess thyroid hormone, which has a synergistic effect with epinephrine or insulin, and therefore, increasing Na^+^/K^+^‐ATPase activity leads to potassium shift into the cells, as seen in our patient. However, without changing the total body potassium level, therefore, during potassium replacement, it should be kept in mind that there is a risk of rebound hyperkalemia.^12^, ^13^ Therefore, when potassium returns to the extracellular space, weakness resolves. Although TPP is usually associated with hypokalemia, TPP may be associated with normokalemia; three cases were reported recently.^14^

Regarding diagnosis, taking a complete history, including family history, drug history, and laboratory examination, including TFTs, blood gas analysis, and urinary analysis for potassium excretion rate, are essential to the differential diagnosis. However, differential diagnoses are divided into two groups: hypokalemic paralysis due to the intracellular shifting of K^+^ or the depletion of potassium in the body. The mainstay of treatment of TPP requires two steps: management of hypokalemia during the attack by potassium replacement (oral or parenteral) and use of non‐selective β‐blockers, which prevent fatal arrhythmia and ensure rapid recovery from muscle weakness.^4^ If potassium should be replaced intravenously, a non‐glucose‐containing fluid must be used to prevent the hypokalemic effect of insulin. In our cases, parenteral potassium replacement was administered.

A low‐carbohydrate diet, non‐selective β‐blockers (Propranolol 40 mg) four times a day, and potassium‐sparing diuretics can be used to prevent attacks until a euthyroid state is achieved. Potassium supplements do not help prevent future attacks and should not be given to patients between episodes.^4^, ^13^ Definitive treatment, however, is to maintain euthyroid status by medical therapy using antithyroid drugs, surgery, or radioactive iodine therapy. Achieving a euthyroid state is very important, preventing future attacks; the recurrence rate reaches 62% within the first three months of diagnosis if the euthyroid status is not achieved. Once the euthyroid state is reached, periodic paralysis attacks usually do not recur. If clinically able, medications such as corticosteroids, non‐steroidal anti‐inflammatory drugs, and other drugs known to trigger attacks should be discontinued during the treatment of TPP.^10^, ^12^

## AUTHOR CONTRIBUTIONS

**Abdulrahman F. Al‐Mashdali:** Conceptualization; data curation; supervision; writing – original draft; writing – review and editing. **Mohammed Alfatih:** Writing – original draft; writing – review and editing. **Waseem Umer:** Writing – original draft; writing – review and editing. **Yassir Eldeeb:** Supervision; validation.

## FUNDING INFORMATION

There are no funders to report for this submission.

## CONFLICT OF INTEREST STATEMENT

The authors have no conflict of interest to declare.

## ETHICS STATEMENT

This case report was approved by the Hamad Medical Corporation's Medical Research Center (Protocol number: MRC‐04‐22‐508).

## CONSENT

Informed written consent was obtained from the patient to publish this case report.

## Data Availability

The datasets used and/or analyzed during the current study are available from the corresponding author on request.
